# Educational and Cognitive Predictors of Pro- and Antisaccadic Performance

**DOI:** 10.3389/fpsyg.2017.02009

**Published:** 2017-11-20

**Authors:** Yaira Chamorro, Mario Treviño, Esmeralda Matute

**Affiliations:** ^1^Laboratorio de Neuropsicología y Neurolingüística, Instituto de Neurociencias, Universidad de Guadalajara, Guadalajara, Mexico; ^2^Laboratorio de Plasticidad Cortical y Aprendizaje Perceptual, Instituto de Neurociencias, Universidad de Guadalajara, Guadalajara, Mexico

**Keywords:** prosaccades, antisaccades, education, reading, adults, neuropsychology

## Abstract

Voluntary gaze control allows people to direct their attention toward selected targets while avoiding distractors. Failure in this ability could be related to dysfunctions in the neural circuits underlying executive functions. Interestingly, recent evidence suggests that factors such as years of schooling and literacy may positively influence goal-directed behavior and inhibitory control. However, we do not yet know whether these factors also have a significant impact on the inhibitory control of oculomotor responses. Using pro- and antisaccadic tasks to assess the behavioral responses of healthy adults, we tested the contribution of years of schooling and reading proficiency to their oculomotor control, while simultaneously analyzing the effects of other individual characteristics related to demographic, cognitive and motor profiles. This approach allowed us to test the hypothesis that schooling factors are closely related to oculomotor performance. Indeed, a regression analysis revealed important contributions of reading speed and intellectual functioning to the choices on both pro- and antisaccadic tasks, while years of schooling, age and block sequence emerged as important predictors of the kinematic properties of eye movements on antisaccadic tasks. Thus, our findings show that years of schooling and reading speed had a strong predictive influence on the oculomotor measures, although age and order of presentation also influenced saccadic performance, as previously reported. Unexpectedly, we found that an indirect measure of intellectual ability also proved to be a good predictor of the control of saccadic movements. The methods and findings of this study will be useful for identifying and breaking down the cognitive and educational components involved in assessing voluntary and automatic responses.

## Introduction

Deciding where to direct one’s gaze plays a crucial role in how people explore the complex world around them. Since people can fixate on only one visual target at a time, they are constantly selecting among different alternatives upon which to focus (Milea et al., 2007). Saccades are rapid eye movements that direct our gaze toward selected visual targets (McPeek and Keller, 2004); however, when distracting stimuli are present, we must also suppress saccades toward unwanted objects.

Experimentally, pro- and antisaccadic tasks are used to assess and quantify specific functional properties of automatic and voluntary saccades. For example, when performing prosaccadic tasks on a computer screen, participants are instructed to move their gaze from a central fixation point to a peripheral target stimulus. Antisaccadic tasks, in contrast, require subjects to avert their gaze from a target stimulus that appears on one side of the screen and direct it in the opposite direction, where there is no stimulus. This voluntary control of saccadic eye movements when performing antisaccadic tasks requires a kind of endogenous control (Munoz and Everling, 2004; Dafoe et al., 2007) that inhibits automatic or ‘inappropriate’ responses to visual inputs and reprograms alternative responses (Crevits and Vandierendock, 2005). Therefore, inhibitory control tasks are commonly used as a measure of the executive functions (i.e., those abilities necessary for purposeful, adaptive and self-directed behavior; Lezak et al., 2012).

Saccadic onset is also influenced by the presence/absence of an initial fixation point. We call the situation in which this point remains visible during the appearance of the target stimulus as the “overlap” condition. Under this condition, the fixation point activates neural processes that maintain the individual’s gaze on the same position until a gaze movement is required (Machado and Rafal, 2000; Bos and Machado, 2013). In contrast, when the fixation point is removed, and a temporal gap introduced prior to the appearance of the target stimulus (the “gap” condition), endogenous (or strategic) control is required. Thus, on antisaccadic tasks, the gap condition demands greater executive control because it elicits saccadic behavior that, instead of being purely “stimulus-driven,” is also based on internal representations (Klein et al., 2002).

It is well-known that the executive functions are subject to the influence of environmental factors (Sabbagh et al., 2006). For example, simply by attending school, subjects confront a complex environment that promotes abstract thinking and improves parallel processing (Castro-Caldas, 2004). Also, school attendance is related to the development of behavior regulation skills (Day et al., 2015) that, in turn, depend on executive functions. Several studies performed with adult populations have reported the effect of years of schooling on executive task performance using such assessment tools as the Trail-Making Test (Hashimoto et al., 2006), category formation, and verbal and non-verbal fluency tests (Spinella and Miley, 2004; Gómez-Pérez and Ostrosky-Solís, 2007). Attending school can be considered a subculture that provides shared knowledge and training in certain skills (Ardila et al., 2010). At school, students must learn and respect rules and schedules, focus and maintain their attention, follow instructions accurately, wait their turn, and achieve long-term goals. These are all skills that require flexible behavioral control and that, obviously, become more complex as people advance from one grade to the next. Thus, in principle, people who attain higher levels of education will acquire not only increasingly complex knowledge, but also more training in abilities related to executive functioning.

Several studies have shown that learning to read influences brain organization (Castro-Caldas, 2004; Ardila et al., 2010) and shapes the development of cognitive functions, including executive ones (Matute et al., 2012). For example, a relation between reading level and components of the executive functions has been evidenced through the study of suppression mechanisms in less-skilled readers (Gernsbacher and Faust, 1991), and failures in access to, and the restrainment of, inhibition in disabled readers when performing working memory tasks have also been reported (Chiappe et al., 2000). It is possible that reading abilities could also impact the executive control of oculomotor responses, since they have a significant effect on visual processing (Castro-Caldas, 2004), impact visual scanning mechanisms (Ostrosky-Solís et al., 1991), and demand a refined control of eye movements in a highly structured visual environment (Radach and Kennedy, 2004).

Although both years of schooling and reading proficiency may well be associated with educational levels, they are not necessarily equivalent. In a study of non-demented elderly Spanish-speakers, Manly et al. (2004) found that reading level has a significant, yet independent, effect on verbal and non-verbal cognitive test performance beyond what years of schooling, age or sex predicted. Those authors sustain that while educational experience does contribute to literacy, individuals have multiple opportunities to enhance their reading ability during their lifetime that may not be reflected in their years of education (Manly et al., 2003).

The aim of the present study was to explore whether the educational factors related to the amount (years of schooling) and quality of education (reading proficiency) are strongly related to executive control over saccadic eye movements. To test this, we analyzed pro- and antisaccadic task performance in adults with different educational levels. We extracted measures related to their saccadic choices (correct/incorrect, and reaction times) and to the kinematic properties of their saccades (i.e., amplitude, average instantaneous speed, average instantaneous acceleration) during significant timing windows. We also tested participants’ precision, speed and comprehension while reading a short story aloud. To probe whether factors like reading proficiency or years of schooling could be strong predictors of the oculomotor measures when studied jointly with other characteristics of individuals, we applied a multivariate regression model with the educative factors and other potential predictors that are known to be related to executive functioning or motor performance; for example, age (Zelazo et al., 2004; Bierre et al., 2016), Intellectual Quotient (IQ) (Lee et al., 2015), right handedness, and manual reaction time (Pirozzolo and Rayner, 1980).

Two sets of saccadic measures were analyzed as dependent variables: choice-related measures (correct responses, errors, anticipations, reaction times); and saccadic kinematics (duration, speed and acceleration). A total of 11 factors were tested as candidate predictors. Four of these were classified as educative factors: years of schooling, reading speed, reading accuracy, and reading comprehension. Age, sex and right handedness were considered demographic factors; while IQ and the total score of *Neuropsi* (a standardized test of general cognitive functioning for adults with low education levels) were used as general intellectual factors. Finally, manual motor reaction time was utilized as a motor factor; and block order presentation as a task control factor.

We hypothesized that among the series of individual characteristics assessed, reading proficiency and years of schooling will have a strong influence on the control of saccadic eye movements, and considered that this influence could be reflected in the execution of those tasks characterized by a high reliance on the executive components of inhibitory control and the programming of alternative responses (i.e., antisaccadic task and overlap conditions).

Our findings provide evidence that reading speed and intellectual abilities are, indeed, strongly related to both pro- and antisaccadic choices, while years of schooling, age and block sequence are important predictors of oculomotor kinematics, especially on antisaccadic tasks. The results of this study help elucidate the relationships among educational and cognitive factors and the control of saccadic eye movements. We consider that quantifying and analyzing the different components involved in oculomotor control are crucial steps in interpreting behavioral variability in participants from heterogeneous backgrounds.

## Materials and Methods

### Participants

The study involved 32 adults (17 men), aged 25–45 years (mean = 32.97 ± 5.92 years), with different educational levels: elementary school (4–6 years of schooling), high school (10–12 years), and college degree or higher (16–18 years). The rationale used in selection was the national mean of years of schooling in Mexico, which is 9.1 (Instituto Nacional de Estadística y Geografía, 2015). We included a similar number of participants who were below (elementary level, *n* = 12), around (high-school level, *n* = 10), and above (college degree or higher, *n* = 10), this mean.

To collect participants’ relevant demographic data and clinical histories, we conducted an initial structured interview. Potential subjects with a history of learning disorders, drug abuse, or neurological or psychiatric conditions were excluded. To homogenize the socioeconomic background of participants, we included only individuals who: (1) lived in Guadalajara, Mexico; (2) began their formal education during their childhood; (3) attended public schools; and, (4) had a job. We applied an additional socioeconomic status questionnaire (SES, Inozemtseva et al., 2016) to confirm the absence of potential differences in socioeconomic status according to educational level (*x*^2^ = 5.39, *p* = 0.25). All subjects signed an informed consent form before participating. The ethics committee of the Instituto de Neurociencias of the Universidad de Guadalajara approved this study, in accordance with the principles of the Helsinki Declaration (Registration number #ET052010-82).

### Materials for Characterizing the Sample

#### Wechsler Adult Intelligence Scale (Wechsler, 2003)

To estimate the IQ, we used the Vocabulary and Block Design sub-tests from the Mexican standardization of the Wechsler Adult Intelligence Scale (WAIS-III) (Wechsler, 2003). Participants with elementary-school level achieved IQ-scores ranging from 70 to 103 (note that this instrument is not standardized for adults with low educational levels). All participants from high school level or higher achieved IQ scores above 80 points. The mean IQ for the entire sample was 97.37 (± 14.85).

#### Neuropsi Test (Ostrosky-Solís et al., 1997)

To confirm that there were no cognitive impairments in the low-educational level group, we assessed normal cognitive functioning using a brief test called ‘Neuropsi,’ a short battery of neuropsychological tests that was developed and standardized with a Spanish-speaking population and that is appropriate for use with such groups. With this battery we quantified a wide spectrum of cognitive functions, including orientation, attention, memory, language, visuo-perceptual abilities, and executive functions (Ostrosky-Solís et al., 1997). For each participant, a total score was calculated (linear sum of all tests). All participants in the sample achieved scores within the range of normality (Neuropsi mean score = 107.91 ± 9.33).

#### Reading Test

To determine participants’ reading performance, we asked them to read aloud a 263-word story in Spanish entitled *Sucedidos* (Galeano, 1994). The following measures were estimated: (1) *reading comprehension*, eight questions about the story (score from 0 to 10 points, sample mean = 5.7 ± 1.8); (2) *reading speed*, the number of words read per minute (sample mean = 120.4 ± 31.3 words); and (3) *reading errors*, percentage of words modified from the original text (sample mean = 3.8 ± 2.9 words).

#### The Edinburg Inventory (Oldfield, 1971)

The Edinburg Inventory (Oldfield, 1971) was used to determine handedness. Results are shown as a percentage of dextral dominance (mean for right-handedness in the sample = 89.32% ± 10.07).

#### Detection Task

A simple visual detection task was designed and applied to record the reaction times of manual responses as a control measure of movement speed. On this task, participants were instructed to press the spacebar on a keyboard with their preferred hand as soon as they noticed the appearance of a circle (1° visual angle) on a computer screen. The task comprised 60 trials (one circle at a time). Each circle appeared for 1,000 ms in the center of the screen and varied in color but not location (i.e., no eye movement was required). Also, inter-stimuli intervals were variable (0.5, 1, 2, or 4 s). We recorded the reaction times for manual responses (in ms) and then calculated the mean reaction time per subject (mean reaction time for the sample = 296 ms ± 7).

### Saccadic Assessment

#### Equipment

Binocular eye movements were recorded with a corneal reflection, non-invasive, eye-tracking system (ET-1750, Tobii Technology AB). This recording system was integrated into a 17-inch computer screen and operated at a sampling rate of 50 Hz with a spatial accuracy of ∼1°.

#### Tasks

Visual stimuli were presented on the eye-tracker screen with participants seated at a distance of 60 cm. We collected and exported the positions of both eyes during each trial using Tobii Studio software (Tobii Technology AB). This software returns a validity code; that is, an estimate of the confidence level at which the position of the eyes has been correctly identified. To avoid low-quality data, all recordings included in this study had ≥80% of samples at or above the accepted level of validity. Each recording session began with a standard 5-dot calibration (Tobii Technology, 2012).

**FIGURE 1:**
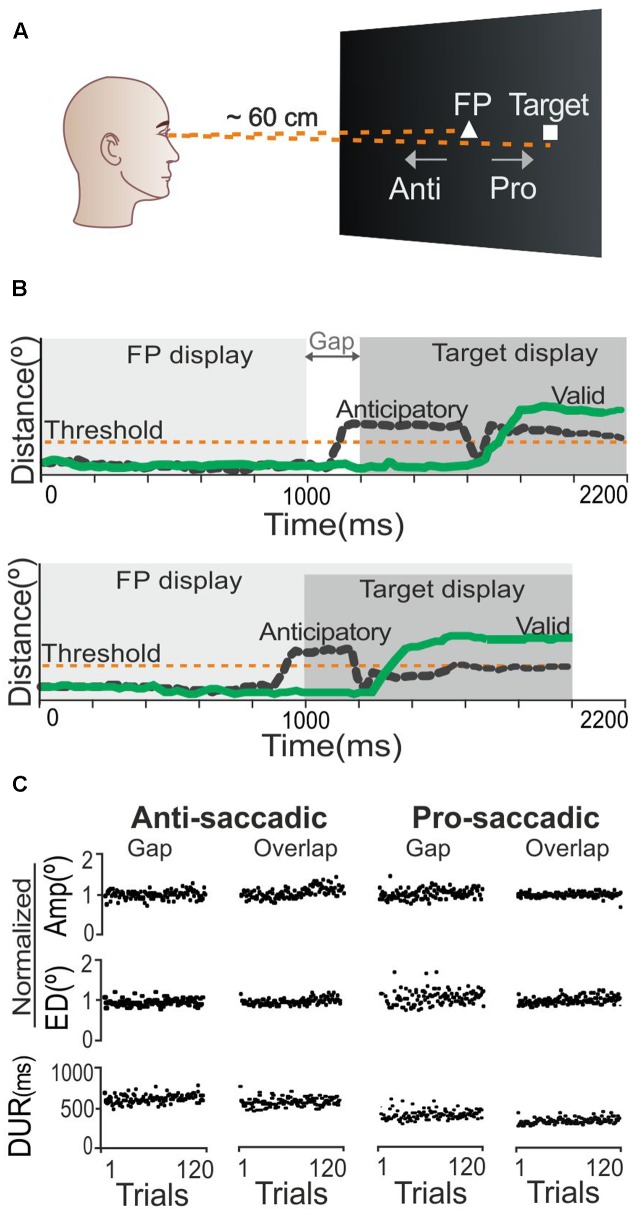
Saccadic task description and control analysis. **(A)** Scheme of visual stimuli including fixation point (FP) and target stimulus location for saccadic tasks. **(B)** Area plots represent the sequence of events in the gap and overlap conditions. Green lines represent the signal of a valid response; black lines represent invalid, anticipatory responses. **(C)** Stable performance throughout all trials was observed for three saccadic parameters: AMP, amplitude (in normalized degrees); ED, error distance (in normalized degrees); and DUR, saccade duration (in ms).

We designed the study in accordance with the experimental oculomotor paradigm described by Klein et al. (2003), such that each participant was required to perform both prosaccadic (gaze movement toward a target stimulus) and antisaccadic (gaze movement away from a target stimulus) tasks (**Figure 1A**), under two stimulus presentation conditions: ‘overlap’ (the fixation point remained on the screen when the target was presented), and ‘gap’ (the fixation point disappeared and the target stimulus was shown 200 ms later) (**Figure 1B**). Each trial began with the presentation of a white triangle (side length = 0.2° visual angle) as the central fixation point. The triangle remained on the screen for 1,000 ms, followed by the target stimulus, which was a white square (size = 0.2° × 0.2°) that appeared 4° away on either the left or right side of the fixation point along the horizontal axis (the side on which it appeared was controlled using random numbers from a binomial distribution). The target remained on the screen for another 1,000 ms. The subsequent trial began 1,000 ms after the target had disappeared. For the prosaccadic tasks, participants were instructed to focus their gaze on the fixation triangle and then to look at the target as soon as it appeared. For the antisaccadic tasks, in contrast, they were instructed *not to look* at the target stimulus, but to shift their gaze in the opposite direction (Klein et al., 2003). Participants performed a total of four blocks of 120 trials each, as follows: (1) prosaccadic task in the gap condition; (2) prosaccadic task in the overlap condition; (3) antisaccadic task in the gap condition; and, (4) antisaccadic task in the overlap condition. To simplify our terminology, we will refer to these blocks as ‘pro-gap,’ ‘pro-overlap,’ ‘anti-gap,’ and ‘anti-overlap,’ respectively. Five practice trials were performed before the first antisaccadic and prosaccadic blocks. To avoid the effect of order presentation, each participant was assigned randomly to one of four different block orders: (1) pro-overlap, anti-overlap, pro-gap, anti-gap; (2) pro-gap, anti-gap, pro-overlap, anti-overlap; (3) anti-overlap, pro-overlap, anti-gap, pro-gap; and, (4) anti-gap, pro-gap, anti-overlap, pro-overlap.

#### Analysis of Eye Movement Data

Eye movement data was exported from the Tobii Studio program and analyzed off-line employing custom-made software to detect saccades using a velocity threshold identification model (I-VT). This algorithm calculated instantaneous velocities and classified the ocular movements as either a fixation (below threshold) or a saccade (above threshold). We used an I-VT of 50°/s (Salvucci and Goldberg, 2000). This saccade detection threshold falls within a valid range (from 30 to 70°/s) reported by other authors (Komogorstev et al., 2009).

For each trial, only the first saccade performed after appearance of the target stimulus was considered. A saccadic response was classified as “valid” when the reaction time was >80 ms, which is the minimum latency required for the visual system to detect and respond to a visual stimulus (Klein et al., 2003). In contrast, responses were classified as “invalid” when the reaction time was <80 ms, or if no saccade was detected. The “valid” saccades were then scored as ‘correct’ or ‘incorrect,’ depending on whether they matched the instruction given. Reaction time from correct and incorrect saccades was extracted. The ‘invalid’ saccades, in turn, were divided into two categories: ‘anticipatory responses’ and ‘omissions.’ We counted the number of valid (correct + incorrect) and invalid (anticipatory + omissions) responses, and then calculated the relative percentages for each one of these categories. In this dataset, correct saccades are complementary to incorrect saccades and anticipatory saccades are complementary to omissions. We discarded the data from experimental blocks in which subjects executed >50% of invalid responses in the total number of trials/block. As a result, eight blocks (four anti-gap, two anti-overlap, one pro-gap, one anti-overlap) were excluded from the complete dataset (consisting of a total of 128 blocks).

Given that our eye-tracker operates with a relatively low sampling rate of 50 Hz, we decided to use broader temporal windows (covering ∼50 frames per second) to extract the kinematic properties of the eye movements. Thus, we calculated the average values of amplitude, velocity and acceleration of the eye movements registered during the presentations of target stimuli and fixation points. More specifically, we defined four temporal windows, as follows: T1: fixation point display (0–1,000 ms); T2: total target display (overlap trials: 1,000–2000 ms; gap trials: 1,200–2,200 ms); T3: the first 200 ms after target appearance (overlap: 1,000–1,200 ms; gap: 1,200–1,400); and, T4: the last 200 ms of target display (overlap: 1,800–2,000; gap: 2,000–2,200).

Before analyzing the influence of the predictors, we tested whether participants’ oculomotor performance was stable during the saccadic tasks, or affected by fatigue. It has been reported that fatigue can produce changes in saccadic parameters such as glissades (slow, drifting eye movements occasionally seen at the end of saccadic eye movements); shorter bursts than appropriate for the size of the intended saccades; and long-duration, among other changes (Bahil and Stark, 1975). For these reasons, we determined three parameters as being indicative of saccadic trajectories and sensitive to learning/fatigue effects: (1) amplitude (in normalized degrees); that is, the sum of the Euclidean distances computed for each gaze-position sample along the path from fixation point to target; (2) error distance (in normalized degrees); that is, the sum of the Euclidean distances calculated between each gaze-position sample and the target position (this measure penalizes saccadic behaviors that keep the gaze position away from the target); and, (3) saccadic duration (in ms); that is, the time elapsed between when the gaze position departed from the fixation point and reached the target position. Results for this control analysis show that the average group value for amplitude, error distance and duration was indeed stable across trials (**Figure 1C**). Moreover, using an analysis of variance for repeated measures, we compared the results from the first 15 trials of each saccadic parameter against those from the last 15 trials. All comparisons produced non-significant results: *F* < 3.45 and *p* > 0.06. This led us to conclude that oculomotor performance was stable across trials, and to rule out possible effects caused by fatigue or learning. Thus, this test allowed us to affirm that additional analyses would be valid.

### Control Analysis

First, we sought to determine whether the eye-movement data from our participants concurred with the principles described in discrimination models (Smith and Radcliff, 2004). As those principles propose, an initial exploration of our data revealed that the saccades with longer reaction times were more likely to be linked to correct responses; a finding that was evident when we collapsed the data from all participants across saccadic tasks (top panel in Supplementary Figure S1A), or considered each saccadic task separately (bottom panels in Supplementary Figure S1A). However, these principles were not fulfilled when analyzing this relation by individual subjects collapsed across condition (Supplementary Figure S1B). This finding highlights the importance of separating the data by condition.

**FIGURE 2:**
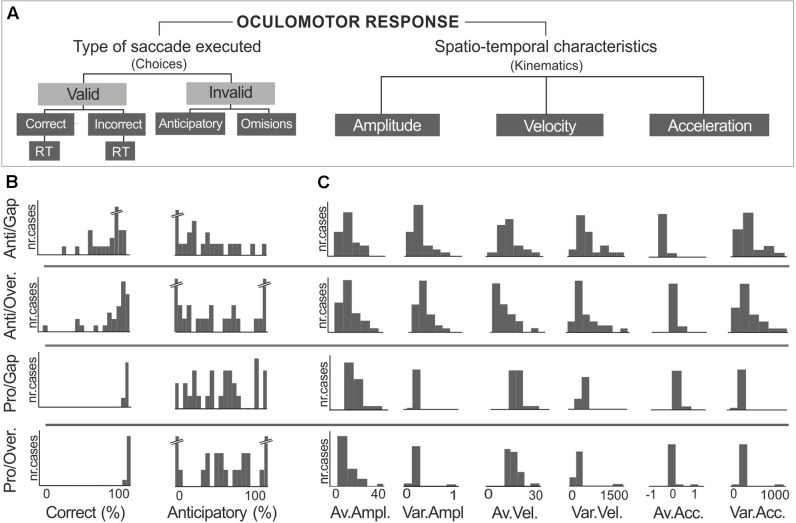
Measures analyzed from oculomotor responses **(A)**. Large dispersion was observed in measures derived from antisaccadic task performance. Examples of distributions are presented for **(B)** percent of correct and anticipatory measures, and **(C)** kinematic properties of amplitude (°), velocity (°/s), and acceleration (°/s^2^).

Having obtained these results, we proceeded to analyze the frequency distribution of saccadic measures related to choices –correct, incorrect, anticipatory saccades and their corresponding reaction times– and to kinematic properties; i.e., amplitude, average instantaneous velocity and acceleration (**Figure 2A**). Results showed dispersion in the correct and anticipatory responses, amplitude, velocity and acceleration, especially on the antisaccadic tasks (**Figures 2B,C**). Thus, a multivariate regression model was applied to identify the sources of this variability in saccadic performance.

### Statistical Analysis

**FIGURE 3:**
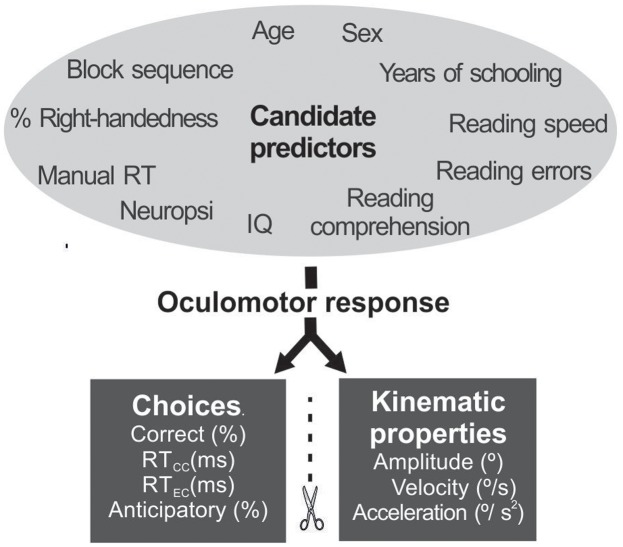
Factors (black letters) and dependent variables (white letters) introduced into the regression model.

The multivariate regression analysis included the four educational factors as predictors: number of years of schooling (6–18), reading speed (number of words read per minute), reading accuracy (% words with errors), and reading comprehension (0–10 score), simultaneously with seven other predictors potentially related to response control: age (25–45 years), sex (male = 1 female = 2), right-handedness (%), IQ (standard score from dual form), Neuropsi score (total raw score), manual motor reaction time (in ms), and order of presentation (pro-gap first = 1, pro-overlap first = 2, anti-gap first = 3, anti-overlap first = 4). Since the predictors selected use distinct scales, we normalized them to a scale of 0–1, before introducing them into the regression model. Two sets of saccadic measures were analyzed as dependent variables: (1) choice-related outcome (% of correct choices, mean reaction time of correct choices -RT_CC_-, RT_CC_ variance, mean reaction time of error choices -RT_EC_-, RT_CC_ variance, and % of anticipatory responses); and, (2) oculomotor kinematic properties (amplitude, average instantaneous velocity, and average instantaneous acceleration) (**Figure 3**).

We used the ‘mvregress’ function with the ordinary multivariate normal maximum likelihood estimation algorithm from MATLAB. Regression coefficients (β) are reported for each predictor. To identify the relevance of the regression coefficients, we established a criterion to determine which of them could be observed due to chance. Although there are formulas that determine the significance of many statistical tests, a much more powerful approach is to compute the empirical probability value for each variable of interest (Mokeichev et al., 2007). This method is widely used for molecular analysis, but it has an important advantage for studies with humans because many assumptions and distributions linked to published formulas may be violated by the heterogeneity observed in empirical datasets like the one we used here. We therefore applied a statistical method based on comparing the actual regression coefficients against surrogate data sets generated by shuffling the predictors (Politis and Romano, 1994; Pipa et al., 2008; Narayanan and Laubach, 2009). In other words, we established the empirical significance of the observed coefficients by comparing them against coefficients that were obtained with randomly permuted predictors (surrogates) in order to test the null hypothesis that the regression coefficients might have been generated by chance.

To identify those predictors that accumulated the strongest associations with saccadic measures, we totaled all the observed regression coefficients that reached significance after the comparison with the surrogate coefficients. Therefore, two sums were performed, one for all the coefficients derived from the analysis of choices: % correct, mean RT_CC_, RT_CC_ variance, mean RT_EC_, RT_EC_ variance, % anticipatory; and the other for all the coefficients from kinematic analysis: means and variances of amplitude, average instantaneous velocity and acceleration in the different temporal windows. Given that there were both positive and negative values, we squared all coefficients before adding them up so that they would not cancel each other out.

A final analysis was conducted to ascertain whether the 11 predictors included in the study could be analyzed in general categories. For this purpose, the predictors were regrouped into five sets: Demographic (sex, age and right handedness), Educative (years of schooling, reading speed, errors, and comprehension), Cognitive (IQ and Neuropsi score), Motor (manual reaction time), and Task control (block sequence). We grouped the predictors by using the MATLAB function ‘pca’ which performs principal component analysis of an input data matrix. We conducted multivariate regression analyses with these five sets instead of the 11 predictors. As in the previous analysis, we determined the observed and surrogate coefficients, compared them to obtain significance, and totaled the significant coefficients obtained in each class to identify the one that returned the strongest associations with oculomotor measures.

## Results

First, we present the results related to choices, followed by the analysis of oculomotor kinematics and, finally, the findings from an integration of the results that was carried out to allow general comparisons.

### Predictors of Saccadic Choices

**FIGURE 4:**
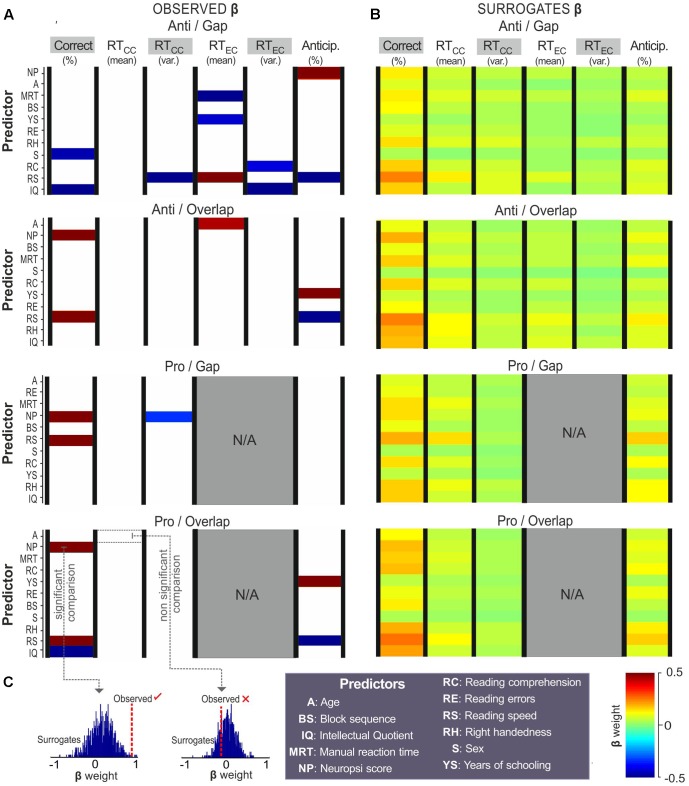
Predictors of choices. Regression coefficients (β) obtained with % correct choices, mean reaction time from correct choices (RT_CC_), RT_CC_ variance, mean reaction time from error choices (RT_EC_), RT_EC_ variance, and % anticipatory saccades. Coefficients from **(A)** observed data and **(B)** surrogates (raw data set to 1,000 permutations) are colored as a function of their weight (color bar). Predictors on the ‘*Y*’-axis are sorted by the total difference between observed and surrogate coefficients. **(C)** *T*-tests were used to compare the observed to the surrogate coefficients, those that reached the level of significance (*p* < 0.05) were colored, those that did not were left blank.

**Figure 4** shows the regression coefficients obtained from the correct choices, reaction times (from correct and error choices) and anticipatory saccades. Coefficient values are represented by color as a function of their weight, as follows: red represents strong positive associations, while dark blue represents strong negative associations (**Figure 4**). Cells left blank represent coefficients that did not reach the level of significance (from the comparison observed vs. surrogate coefficients).

As can be seen, reading speed and Neuropsi scores showed strong associations with saccadic choices on all four tasks. Years of schooling and IQ also returned strong associations with several choices. Higher reading speed was related to a greater number of correct choices on the anti-overlap (β = 0.85, *p* = 0.01), pro-gap (β = 1, *p* = 0.02), and pro-overlap tasks (β = 0.86, *p* = 0.01). It was also associated with a lower variance in reaction time (β = -0.92, *p* = 0.02) and higher error reaction times in the anti-gap block condition (β = 0.75, *p* = 0.01). Reading speed was associated with fewer anticipatory responses on the anti-gap (β = -0.87, *p* = 0.03), anti-overlap (β = -0.75, *p* = 0.04), and pro-overlap tasks (β = -0.67, *p* = 0.04).

A higher Neuropsi score was also related to a greater number of correct responses on the anti-overlap (β = 0.75, *p* = 0.01), pro-gap (β = 0.91, *p* = 0.01), and pro-overlap tasks (β = 0.65, *p* = 0.03). It was also moderately associated with a shorter variance in reaction time on the pro-gap tasks (β = -0.32, *p* = 0.04) and a greater number of anticipatory responses on the anti-gap task (β = 0.84, *p* = 0.03).

Intellectual Quotient was negatively related to the percentage of correct choices on the anti-gap (β = -0.81, *p* = 0.04), pro-gap (β = -0.94, *p* = 0.02) and pro-overlap tasks (β = -0.63, *p* = 0.02); and to the error reaction time, though only on the anti-gap task (β = -0.68, *p* = 0.02).

Years of schooling was positively associated with anticipatory responses on the anti-overlap (β = 0.83, *p* < 0.01) and pro-overlap tasks (β = 0.73, *p* < 0.01); and negatively associated with error reaction time on the anti-gap task (β = -0.42, *p* = 0.03).

Manual reaction time, sex and reading comprehension showed isolated associations with error reaction time (β = -0.72, *p* = 0.04), % of correct choices (β = -0.46, *p* = 0.04), and error reaction time variance (β = -0.41, *p* = 0.04), respectively, all on the anti-gap task.

In summary, in terms of choices, reading speed and Neuropsi were determined to be strong predictors of correct anticipatory responses, and reaction times on both pro- and antisaccadic tasks. Years of schooling and IQ were also associated –though in the opposite direction– to correct and anticipatory responses. In the anti-gap block, other predictors showed strong influences, such as manual reaction time, sex and reading comprehension.

### Predictors for Oculomotor Kinematics

To analyze whether the candidate predictors had an impact on the kinematic properties of the eye movements recorded during target stimulus display and fixation point display, we conducted a similar regression analysis using the same predictors, and three measures: amplitude, average instantaneous velocity, and average instantaneous acceleration (means and variances). We analyzed separately the kinematic properties of correct, incorrect and anticipatory saccades, in order to determine whether the type of choice influenced the kinematics output.

Kinematics were extracted from relevant temporal windows to confirm that the relation between predictors and oculomotor measures did not vary along oculomotor trajectories. Thus, each regression was analyzed during the following phases: fixation point display (T1); target display (T2); in the first 200 ms of target appearance (T3); and during the final 200 ms of target display (T4). For the correct and incorrect saccades, we focused analysis on target display (T2, T3, and T4) because the saccadic kinematics analyzed during fixation point display (T1) correspond to anticipatory responses.

**FIGURE 5:**
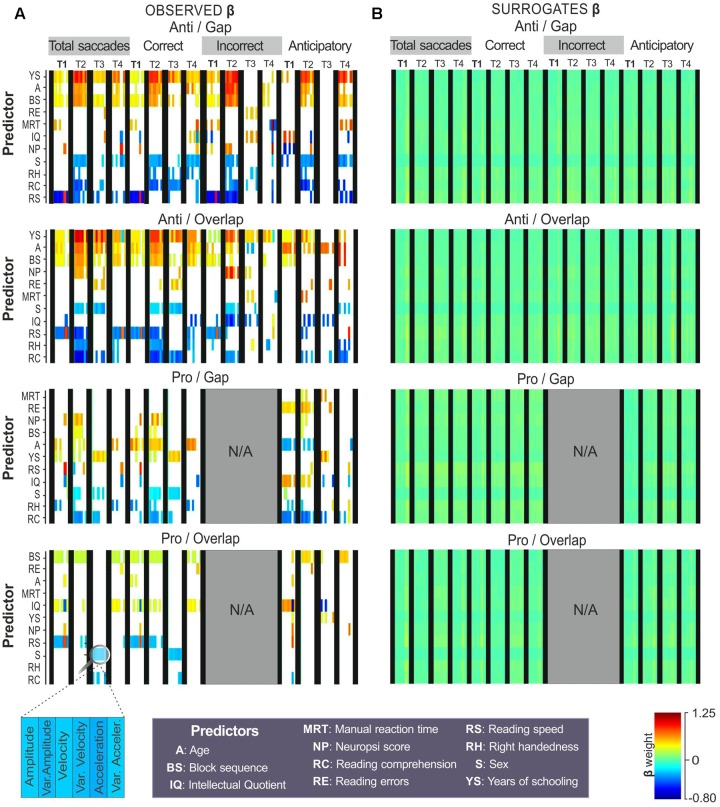
Predictors of oculomotor kinematics. Regression coefficients (β) obtained with amplitude, average instantaneous velocity and acceleration during four temporal windows: fixation point display (T1), total target display (T2), first 200 ms of target appearance (T3), and final 200 ms of target display (T4). Coefficients from **(A)** observed data and **(B)** surrogates are colored as a function of their weight (color bar). Predictors are sorted by the difference between observed and surrogate coefficients (*Y*-axis). *T*-tests were used to compare the observed to the surrogate coefficients, those that reached the level of significance (*p* < 0.05) were colored, those that did not were left blank.

The coefficients that were seen to reach the level of significance in the *t*-test comparison with surrogate coefficients, are colored as a function of their weight on the color map in **Figure 5**. As in **Figure 4**, blank cells correspond to coefficients with non-significant comparisons. Since this analysis comprises many saccadic measures, the color map helped visualize patterns of significant associations between the predictors and the kinematic measures across the different temporal windows.

On the antisaccadic tasks, years of schooling, age, and block sequence showed strong, positive associations with kinematics; while reading speed, reading comprehension and sex turned out to be negatively associated with oculomotor kinematics.

On both anti-gap and overlap tasks, the factor years of schooling was positively associated with kinematics, particularly during stimulus display (T2) from total (β-weights ranging from 0.50 to 0.94) and correct responses (β-weights from 0.45 to 0.87), as well as from incorrect (β-weights from 0.64 to 0.99) and anticipatory responses (β-weights from 0.47 to 0.89) on the anti-gap task.

Age and block sequence showed positive associations with kinematics on both antisaccadic tasks, especially during stimulus appearance (T2) in total (β-weights from 0.34 to 0.73), correct (β-weights from 0.48 to 0.73) and incorrect responses (β-weights from 0.35 to 0.87).

Higher reading speed was related to lower values in antisaccadic kinematics during stimulus (T2) from total (β-weights from -0.49 to -0.72), correct (β-weights from -0.52 to -0.59) and incorrect responses (β-weights from -0.67 to -0.86). Higher reading comprehension was associated with lower values in antisaccadic kinematics during stimulus display (T2) and the first 200 ms of stimulus appearance (T3) from total (β-weights from -0.50 to -0.60), correct (β-weights from -0.53 to -0.58) and incorrect responses (β-weights from -0.38 to -0.52). Sex returned moderate associations with kinematics during T2 and T3 of the total (β-weights from -0.27 to -0.40), correct (β-weights from -0.23 to -0.45) and incorrect responses (β-weights from -0.22 to -0.43).

With respect to the prosaccadic tasks, the predictors showed moderate-to-low associations with kinematic properties. No regression analysis was conducted for the incorrect responses from the prosaccadic tasks (gap and overlap) due to their infrequency. In the correct responses on the pro-gap task, age showed positive associations with kinematic properties, particularly during stimulus display (T2), and the last ms of stimulus appearance-T4 (β-weights from 0.33 to 0.69); sex (β-weights from -0.24 to -0.22) and reading comprehension (β-weights from -0.18 to -0.43) were negatively related to kinematics on stimulus display (T2). In the anticipatory responses of pro-gap, reading errors (β-weights from 0.45 to 0.50) and IQ (β-weights from 0.58 to 0.63) showed positive associations with kinematics in fixation and stimulus display. Age (β-weights from -0.34 to -0.38) and reading comprehension (β-weights from -0.47 to -0.48) were negatively related to kinematics in these same temporal windows.

On the pro-overlap task, block sequence, IQ and reading speed were the predictors that showed consistent, though weak, associations with kinematics across the different temporal windows. Block sequence returned moderate associations during T2 on total (β-weights from 0.24 to 0.28) and correct responses (β-weights from 0.24 to 0.28), and during T2 and T4 in anticipatory responses (β-weights from 0.46 to 0.49). IQ showed positive associations with kinematics during the last ms of stimulus appearance in total (β-weights from 0.39 to 0.42) and correct responses (β-weights from 0.40 to 0.44), as well as with anticipatory responses performed during fixation display (β-weights from 0.60 to 0.65).

In summary, several predictors proved to be related to kinematics on the antisaccadic tasks: years of schooling, age, block sequence, reading speed, reading comprehension, and sex. On the prosaccadic task, kinematics showed moderate associations with block sequence, IQ, reading speed and age.

### Integrating Results from Choices and Kinematics

**FIGURE 6:**
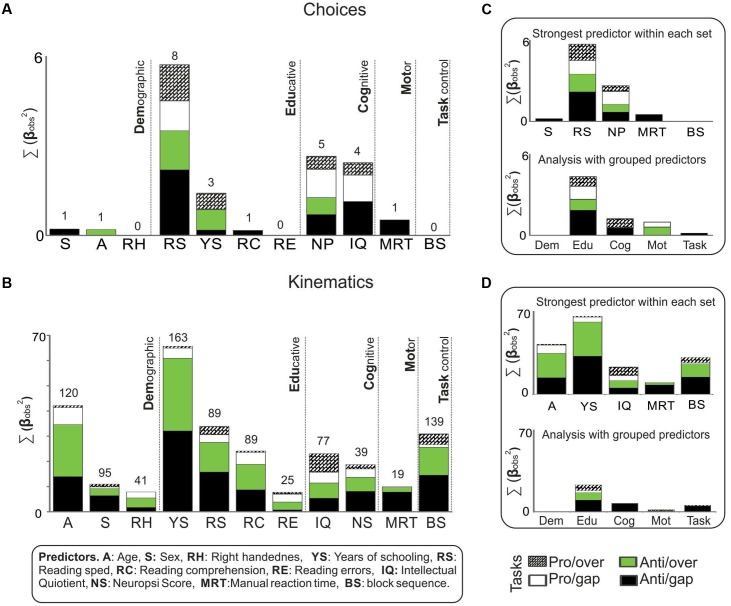
Predictors strength. Bars represent, by predictor, the total sum of the weights of significant observed coefficients (β_obs_) from the analysis of **(A)** choices and **(B)** kinematics, on the four saccadic tasks. All coefficients that reached significance after comparing them to surrogates were squared and summed. The total number of significant β_obs_ summed are presented above each bar. A parallel regression analysis was conducted with components that regrouped the predictors into five sets: Demographic, Educative, Cognitive, Motor, and Task control. The coefficients that resulted from **(C)** choices and **(D)** kinematics were squared and summed. The grouping strategy resembles the main findings from testing predictors individually.

To summarize the main findings of this study, we proceeded to identify the predictor that accumulated the strongest associations with saccadic choices and kinematics. With this goal in mind, we totaled each predictor, and all the observed regression coefficients that reached significance after comparison with the surrogate coefficients. Two sums were performed; in the first, we included all the coefficients derived from the analysis of choice measures (**Figure 6A**), while the second considered all the coefficients from kinematic analysis (**Figure 6B**). We also calculated the sum of of non-significant coefficients for complementary information (Supplementary Figure S2).

We found that reading speed was the predictor that achieved the strongest associations with choice measures and the greatest number of significant coefficients. Neuropsi scores, IQ and years of schooling showed more moderate associations. Age, sex, reading comprehension, and manual reaction time accumulated fewer associations and, significantly, only with antisaccadic measures. Right handedness, reading errors and block sequence failed to return any significant association with choices.

In the case of kinematic measures, several predictors accumulated significant associations. Years of schooling was the one with the strongest associations, as well as the greatest number of significant coefficients. Age, reading speed and block sequence were the other predictors that had strong associations. Reading speed, reading comprehension, IQ, and Neuropsi scores returned more moderate associations. Sex, right handedness, reading errors and manual reaction time showed the weakest associations. It is noteworthy that the significant associations were registered primarily on the antisaccadic tasks.

Finally, we analyzed the possibility of reducing the predictors into general categories, or sets of predictors. We thus regrouped all the predictors into five sets: Demographic, Educative, Cognitive, Motor, and Task control, and proceeded to perform the regression analysis with these five categories instead of the 11 individual predictors. Once the observed and surrogate coefficients were determined, they were compared. The coefficients that reached significance in this comparison were then totaled. Results are presented in **Figures 6C,D**. As this figure shows, the set of educative predictors showed the strongest association with both the choices and the kinematics analysis. It was followed by the cognitive set. In the case of choices, the educative predictors showed the strongest associations with measures from both antisaccadic and prosaccadic tasks, but the amount was particularly large in the case of anti-gap choices. With respect to kinematics, the educative set seemed to be related to kinematics, especially antisaccadic kinematics.

## Discussion

The present study was designed to identify, and then quantify, the effect of educative factors on saccadic eye movement control. Our hypothesis was that if saccadic eye movement control –specifically, antisaccadic task performance– is indeed a measure of executive function, then it would be related to education and reading proficiency, since schooling-related factors have been shown to affect executive control over behavioral tasks (Ostrosky-Solis et al., 2004).

When applied to the saccadic choices, the regression analysis revealed an important relation to reading speed and years of schooling, as well as to Neuropsi scores and IQ. When applied to kinematics, it showed associations with years of schooling, age and block sequence on the antisaccadic tasks, with only moderate and weak associations for the prosaccadic tasks.

In relation to reading speed, we found that it was related to more accurate performance on both prosaccadic and antisaccadic tasks (associations with higher correct choices and lower anticipatory responses). These results suggest that being a fast, skilled reader is related to experience in terms of programming when to begin an eye movement and where to assign the subsequent fixation, just as poor reading in children has been linked to the impaired execution of antisaccades (Biscaldi, 2000; Lukasova et al., 2016). Although a relation between oculomotor factors and dyslexia (Biscaldi, 2000), and executive functioning –mainly working memory and general processing speed, and word reading and comprehension in children (Christopher et al., 2012)– have been pointed out, the direction of their interplay in reading disabilities is not yet clear, and our study also failed to clarify this issue. Given the nature of the regression analysis performed in this study, it is not possible to determine whether reading speed is a cause, or a consequence, of the variability in the oculomotor control of eye movements. Hence, this issue needs to be addressed in a future study designed expressly for that purpose.

In the case of shallow orthographic systems, such as Spanish, reading speed has been identified as the most sensitive measure of reading proficiency, even surpassing accuracy (Simos et al., 2013), to the extent that low reading speed is more characteristic of poor Spanish-speaking children than the frequency of reading errors (Matute et al., 2000; Escribano, 2007). This issue may explain why the other reading measures were not found to be strongly related to saccadic performance in Spanish readers. Furthermore, the fact that reading speed was related to performance on both types of tasks (pro- and antisaccadic), for both fixation conditions (gap and overlap), suggests that this factor is not related exclusively to the inhibition of oculomotor automatic responses, but also to the execution of visually triggered saccades.

Reading speed was negatively associated with oculomotor kinematics on the antisaccadic tasks and –though moderately– on the prosaccadic ones during stimulus display. Thus, higher reading speeds can be related to lower kinematic values, suggesting that lower variability in kinematics may be observed in fast readers. In one of the earliest studies of saccadic dynamics, Yarbus (1967) analyzed whether kinematics (saccadic duration and speed) depends on the subject’s will. The conclusion was that adults cannot voluntarily change the character of a saccade, but only the duration and frequency of fixations. Therefore, it could be expected that kinematic properties, unlike choices, will not be affected by the predictors tested in our study through voluntary control. Nevertheless, we found that reading speed returned strong associations across the temporal windows in relation to kinematics; especially on the antisaccadic tasks and, moderately, on the prosaccadic ones. Although adults may voluntarily manipulate how fast they read, certain automatic cognitive processes underlie fast reading, such as the use of parafoveal information to anticipate word identification, or “bottom-up” processes that enable fast readers to gain visual access to lexical representations and so identify words more quickly (Jordan et al., 2016). Our results suggest that automatic processes during reading might be related to the automatic programming of eye movement kinematics.

Among the other predictors analyzed, Neuropsi scores, a measure of general cognitive functioning, returned strong associations with saccadic choices. Here, higher scores were related to a greater number of correct responses on the anti- and prosaccadic tasks, and to lower variability in the reaction times of correct responses on pro-gap tasks. Therefore, general cognitive functioning seems to be closely related to saccadic eye movement control; a finding that agrees with the assumption that intellectual behavior relies on the individual’s ability to process task-relevant information while simultaneously inhibiting task-irrelevant information and responses (Lee et al., 2015). The relation of general cognitive functioning over saccadic performance could be of interest for clinical purposes. For example, the assessment of voluntary eye movements could be used not only to restore communication with physically challenged patients, but also to assess their cognitive level.

In relation to oculomotor kinematics, significant associations were observed mostly during target display (temporal window T2), suggesting that relations did not change along oculomotor trajectories. Several predictors showed strong associations with kinematics on the antisaccadic tasks, and moderately on the prosaccadic ones. In fact, the kinematics on prosaccadic tasks seemed to be mildly related to the predictors analyzed in our study. It is to be expected that the metrics of antisaccadic trials will be more variable than those of prosaccadic ones, since the eye movements are not directed to any visible landing point (Munoz and Everling, 2004). According to our results, part of the variability in the kinematic properties seems to be related to age, block sequence and years of schooling. In relation to age, although we did not include participants over 45 in our study in order to avoid the effects of aging as reported by Klein et al. (2005), and Chen and Machado (2016), the antisaccadic and prosaccadic kinematics were positively related to age, whereas the choices measures were not. Raemaekers et al. (2006) reported that mid-adults (30–55 years old) show similar performance on pro- and antisaccadic tasks to that of younger adults, but with an increased activation of the frontal areas involved in the programming of voluntary eye movements (frontal eye fields) (Olk et al., 2006). The kinematic variability linked to age that we found could be related to the compensatory activation of such frontal regions, as reported by Raemaekers et al. (2006). Block sequence also showed moderate, but consistent, associations with kinematics on both anti- and prosaccadic tasks. It would be important to consider this factor in studies involving oculomotor kinematics.

Years of schooling showed unexpected positive associations with kinematics (higher means, higher variances), and with anticipatory errors on the pro- and anti-overlap tasks. The use of years of schooling to represent a direct effect of experience on cognition could be considered problematic when analyzing adults from diverse social backgrounds, since not all adults who complete a certain school grade necessarily attain the associated academic achievement (Manly et al., 2003). Other factors, such as ceiling effects, restrict the interpretation of results; for example, Mirsky et al. (2011) found a differential association between antisaccadic performance and executive functions in older adults as a function of the level of education (measured according to the number of years of schooling). In their study, positive associations were observed in the low-level group, but no such associations were seen in the high-level groups. Also, as described above, reading speed had a positive relation to correct responses and a negative association with anticipatory responses on both prosaccadic and antisaccadic tasks. Our results suggest that, as Manly et al. (2003,^ 2004^) reported, literacy captures aspects of educational experience that are not accounted for by years of education alone. This offers support for the proposal to use other indexes of educational quality –such as reading proficiency– instead of depending exclusively on the number of years of schooling. Thus, it could be that reading proficiency has an independent association with oculomotor control beyond that related to years of schooling.

Intellectual Quotient, the measure of intellectual ability employed in our study, also showed an inverse association pattern with saccadic choices; i.e., higher IQ scores were related to lower numbers of correct responses and shorter variances in error reaction times on the anti-gap tasks. The lack of norms for populations with different levels of schooling might affect the relationships observed between IQ and performance on other tasks. The fact that we include individuals with low IQ levels (those from the elementary school level) may well also have an impact on the strong correlations observed in our study. On the one hand, most studies concerning the relationship between cognitive abilities and IQ have not included low-IQ participants in their samples; on the other, Detterman and Daniel (1989) reported that average correlations between cognitive tasks and IQ are observed when only high-IQ participants are studied, but if the same correlations are computed for low-IQ adults, they are about twice as large. Those authors conclude that studies of individual differences in cognition that do not include a proportional representation of low-IQ subjects are difficult to interpret. We did not discard adults with low IQ because they had an educational level not included in the Wechsler scales standardization; instead, we ensured that their cognitive functioning was within the normality ranges using a standardized instrument. Nevertheless, Neuropsi and IQ scores showed opposite associations with correct responses (positive for Neuropsi, negative for IQ). Although both of these scores are considered indexes of general cognitive functioning, IQ is related to intelligence, while Neuropsi covers a wide range of cognitive domains. The different perspectives from which these two measures were designed could explain the different results they generate.

In an attempt to limit socioeconomic discrepancies in this study, we included only adults who lived in the same city, had attended public schools, began their education in childhood, and were economically active; however, it is clear that this issue needs to be addressed specifically in future studies. The effort to control for these issues reduced the number of participants, so it is clear that future studies with larger samples would strengthen the statistical power of our results.

Seeking to reduce the number of comparisons conducted, we analyzed the outcome of reducing all 11 predictors to more generic categories, and then performed the regression analysis with five categories instead of 11. The findings generated by the final analysis of our study showed that the results from the grouping strategy resemble the main findings based on testing predictors individually, since the strongest predictor match in both strategies was with the educative factors, followed by the cognitive ones. Thus, another possibility for further studies that wish to avoid dealing with multiple testing issues is to use groups of predictors instead of testing many predictors individually. However, the interpretation of the results obtained collapsing predictors in sets, would not be exactly the same as those from the individual testing. In fact, of the educative factors, reading speed was the predictor with the strongest associations in choice measures, whereas years of schooling had the strongest associations for saccadic kinematics. These results are not obvious when making sets of predictors. Both analyses are valuable; the choice of one of them will depend on the main objective of the study.

To summarize, the results of this study suggest that, among different individual characteristics, educative factors followed by intellectual functioning factors are closely related to pro- and antisaccadic choices. The kinematic properties of saccades, especially antisaccades, are also related to educative factors and, though to a lesser extent, age and task sequence. Nonetheless, a difference within educative factors was also evident, since reading speed was the one more related to choice measures, whereas number of years of schooling was closely associated to the kinematic ones.

According to our results, variations in saccadic measures may reflect reading proficiency and general cognitive functioning, and not only the effect of age as reported in developmental studies (Munoz et al., 1998; Klein et al., 2003; Pratt et al., 2006), task manipulations (Spering et al., 2008), or neurological dysfunction, as reported previously for clinical populations (LeVasseur et al., 2001; Munoz et al., 2003; Machado and Rafal, 2004; Karatekin, 2006; Rivaud-Pechoux et al., 2007; Levy et al., 2008). Taking educational and cognitive factors into account in studies of this kind will shed light on the application and interpretation of eye-movement assessment in the fields of basic or clinical research.

## Author Contributions

YC: Substantial contribution to the conception of the work, to data acquisition, analysis and interpretation; drafting; final approval of the version to be published; agreement to be accountable for all aspects of the work by ensuring that questions related to the accuracy or integrity of any part of same have been appropriately investigated and resolved. MT: Substantial contribution to data analysis and interpretation; revising the draft and contents for important intellectual contributions; final approval of the version to be published; agreement to be accountable for all aspects of the work by ensuring that questions related to the accuracy or integrity of any part of same have been appropriately investigated and resolved. EM: Group leader, substantial contribution to the conception of the work and to data analysis and interpretation; revising the draft and contents for important intellectual contributions; final approval of the version to be published; agreement to be accountable for all aspects of the work by ensuring that questions related to the accuracy or integrity of any part of same have been appropriately investigated and resolved.

## Conflict of Interest Statement

The authors declare that the research was conducted in the absence of any commercial or financial relationships that could be construed as a potential conflict of interest.
