# Supplementary figures and images for: Educational and Cognitive Predictors of Pro- and Antisaccadic Performance
Source: Front Psychol. 2017 Nov 20;8:2009. doi: 10.3389/fpsyg.2017.02009 (PMC5701939; doi:10.3389/fpsyg.2017.02009)

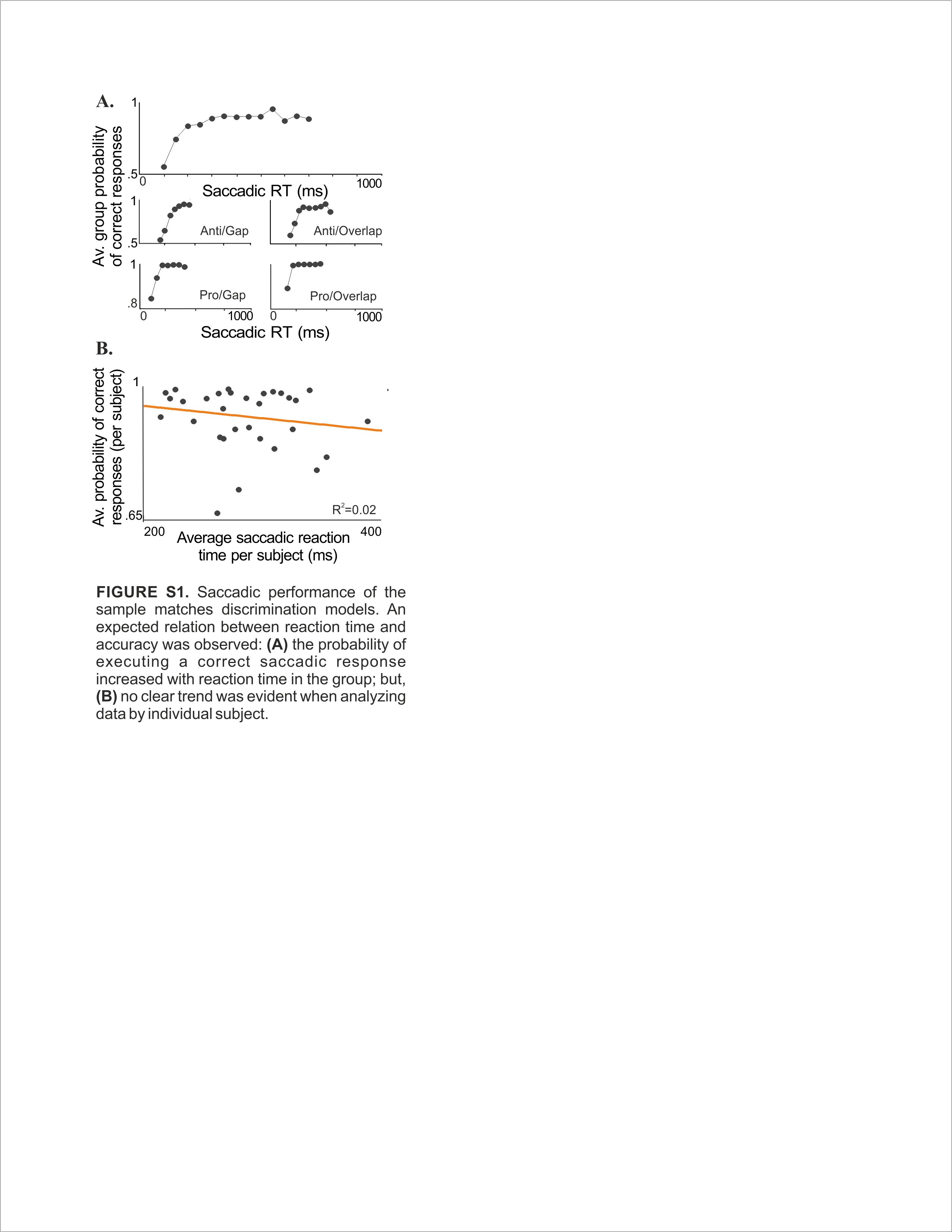

Supplement: Supplementary file 1 [file Image_1.jpg]

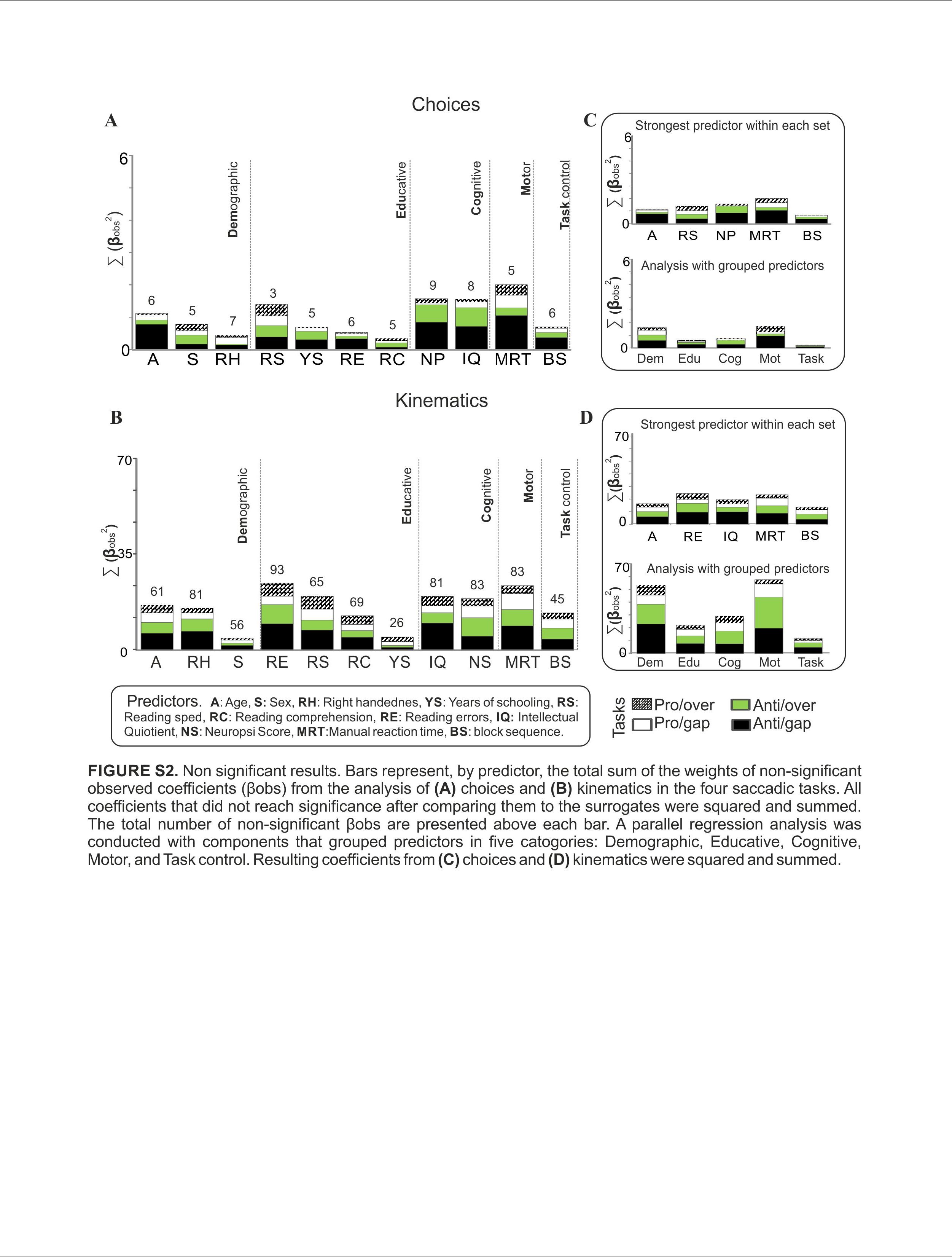

Supplement: Supplementary file 2 [file Image_2.jpg]
